# The effect of sexual abuse and dissociation on suicide attempt

**DOI:** 10.1186/s12888-021-03662-9

**Published:** 2022-01-10

**Authors:** Silje Støle Brokke, Thomas Bjerregaard Bertelsen, Nils Inge Landrø, Vegard Øksendal Haaland

**Affiliations:** 1 Department of Psychiatry, Sørlandet Hospital HF, Po box 416, N-4604 Kristiansand, Norway; 2 Clinical Neuroscience Research Group, Department of Psychology, Faculty of Social Sciences, University of Oslo, Oslo, Norway; 3 Department for Child and Adolescent Mental Health, Sørlandet Hospital, Kristiansand, Norway; 4 Division of Mental Health, Sørlandet Hospital, Kristiansand, Norway

**Keywords:** Sexual abuse, Trauma, Dissociation, Suicidal behavior, Suicide attempt

## Abstract

**Background:**

Suicide attempt is the most predictive risk factor of suicide. Trauma – especially sexual abuse – is a risk factor for suicide attempt and suicide. A common reaction to sexual abuse is dissociation. Higher levels of dissociation are linked to self-harm, suicide ideation, and suicide attempt, but the role of dissociation in suicidal behavior is unclear.

**Methods:**

In this naturalistic study, ninety-seven acute psychiatric patients with suicidal ideation, of whom 32 had experienced sexual abuse, were included. Suicidal behaviour was assessed with The Columbia suicide history form (CSHF). The Brief trauma questionnaire (BTQ) was used to identify sexual abuse. Dissociative symptoms were assessed with Dissociative experiences scale (DES).

**Results:**

Patients who had experienced sexual abuse reported higher levels of dissociation and were younger at onset of suicidal thoughts, more likely to self-harm, and more likely to have attempted suicide; and they had made more suicide attempts. Mediation analysis found dissociative experiences to significantly mediate a substantive proportion of the relationship between sexual abuse and number of suicide attempts (indirect effects = 0.17, 95% CI = 0.05, 0.28, proportion mediated = 68%). Dissociative experiences significantly mediated the role of sexual abuse as a predictor of being in the patient group with more than four suicide attempts (indirect effects = 0.11, 95% CI = 0.02, 0.19, proportion mediated = 34%).

**Conclusion:**

The results illustrate the importance of assessment and treatment of sexual abuse and trauma-related symptoms such as dissociation in suicide prevention. Dissociation can be a contributing factor to why some people act on their suicidal thoughts.

**Supplementary Information:**

The online version contains supplementary material available at 10.1186/s12888-021-03662-9.

## Background

Suicide accounts for more than 700,000 deaths yearly worldwide [1]. Suicidal behavior can include different levels of severity from suicide thoughts to death by suicide [2]. Traditional risk factors like depression, hopelessness, most psychiatric disorders, and impulsivity can predict suicide ideation, but such factors poorly predict suicide attempts among suicide ideators. In an ideation-to-action framework to suicide, the progression from suicide ideation to lethal suicidal attempts can be understood as a distinct process. This process can include factors that diminish fear of pain, injury, and death which can increase a person’s capability to attempt suicide [3].

People who are exposed to early traumatic events are at increased risk of attempting suicide compared to the general population [4]. Sexual abuse, especially in childhood, has consistently been associated with suicidal behavior [5]. An early Australian study of 183 young people found the suicide rate of people reporting sexual abuse to be 10.7–13.0 times the national rates at 9 years after inclusion in the study. Of those who reported sexual abuse, 43% reported suicide ideation and 32% reported suicide attempts [6]. Recent meta-analyses have confirmed that childhood sexual abuse is an important risk factor for suicide attempts [7, 8].

A large body of research has identified a relationship between potentially traumatizing events and dissociative symptoms [9]. Dissociation can be understood as a form of detachment and can include depersonalization, derealization, amnesia, fugue states, and identity disorders [10]. Dissociation can include a disconnection from the body that can reduce fear and pain associated with harming the body that can make suicide attempt possible [11].

Several studies have found dissociation to be a predictor of suicide attempts [12–15]. Dissociation has been found to differentiate individuals with a history of suicide attempt from those with suicide ideation alone [11]. Research show that higher levels of dissociation can be an important mediating factor, regardless of psychiatric disorders in the development of self harm and suicide attempt [16].

Dermirkol and colleagues found that childhood maltreatment is a strong predictor of suicide attempts and that “psychache” and dissociation play mediator roles in this relationship [17]. They found dissociation to have a full mediator role in the effect of emotional abuse and physical abuse on suicide attempt and partial mediator roles in the effect of sexual abuse and physical neglect on suicide attempt. However, one limitation of their study was its wide age range, as the effect of the trauma may decrease with age.

A systematic review of the literature confirms there is robust evidence for a mediating role of dissociation in trauma and non-suicidal self-injury, but the literature is lacking evidence of the mediating role of dissociation in suicide ideation and completed suicides [18]. There are, to our knowledge, no studies of the association between dissociation and sexual abuse in suicide risk that differentiates between single- and multi-suicide attempters.

In this study, acute psychiatric patients at suicide risk who had experienced sexual abuse were compared to patients with suicide risk who had not experienced sexual abuse. The role of dissociation in the association between sexual abuse and suicide attempts was thereby analyzed which has the potential to fill a knowledge gap. We predicted that (1) patients having experienced sexual abuse would be more likely to have a history of suicide attempt, (2) they would report higher levels of dissociation, and (3) dissociation would have a mediating effect on the association between sexual abuse and suicide attempts.

## Methods

### Participants

The participants included were acute psychiatric patients referred to the crisis resolution team at Sørlandet Hospital in Southern Norway. The crisis resolution teams aim to help people experiencing mental health crises, usually related to suicide risk and acute mental illness. The inclusion period was from May 2014 to August 2017. Patients aged between 18 and 65 years referred to the mental health service for suicide risk were included. Suicide risk could include suicide thoughts, suicide threats, suicidal behavior, and suicide attempts. Three hundred sixty-seven patients were asked to participate, 227 patients agreed to participate and 97 completed all measurements included for the analysis in this study. When comparing the patients who agreed to participate with the patient group that did not want to participate there is no significant difference in age, gender or depression. The exclusion criteria were severe substance abuse and the inability to read, speak, or write Norwegian. The Regional Committee for Medical and Health Research Ethics (2013/1664/REK sør øst D) approved the study.

### Measures

#### Brief trauma questionnaire (BTQ)

The brief trauma questionnaire (BTQ) [19] is a 10-item self-report questionnaire used to assess experiences of traumatic events. In the present study, the BTQ was included to assess unwanted sexual contact and this experience was labeled “sexual abuse.” The question was asked, “Has anyone ever made or pressed you into having some type of unwanted sexual contact? By “sexual contact,” we mean any contact with someone else and your private parts or between you and somebody else’s private parts. Have this ever happened to you? *Yes* or *no*.” Sexual abuse was coded as a dichotomous variable (0 = not present, 1 = present). The psychometric data for the BTQ is somewhat limited, but interrater reliability has been shown to be good for all the primary trauma categories and criterion validity, with associations found consistently between PTSD-symptom severity and BTQ-measured trauma [20].

#### Dissociative experiences scale (DES)

The dissociative experiences scale (DES) is a 28-item self-report form used to measure dissociative symptoms [21]. The questionnaire is the most widely used self-report measure of dissociation and has been found to give reliable measures of dissociation in clinical and non-clinical populations [21]. In the present study, the total scale score (range 0–100) was calculated by averaging across all 28 items. Pathological dissociation was identified by using 8 items from the DES, DES-T [22].

#### Columbia suicide history form (CSHF)

The Columbia suicide history form (CSHF) [23] was selected for this study to assess the severity of suicidal behavior and, specifically, an individual’s suicide-attempt history. The CSHF is structured as a screening interview, with 5 questions on suicide ideation, 7 questions on intensity of ideation, 6 questions on suicidal behavior, and 2 questions on lethality evaluations of actual attempts. The interview covers both suicidal behavior during the previous month and lifetime history of suicidal behavior for all the different questions. Suicide attempts are further categorized by the first, latest, and most deadly attempts. Most questions in the interview are *yes or no*-based, some questions are age-based, and some questions ask for the number of occurrences of suicidal behavior. The CSHF has shown good convergent and divergent validity with other multi-informant suicidal ideation and behavior scales, and it has been validated as a suitable assessment of suicidal ideation and suicidal behavior in both clinical and research settings [24].

#### Montgomery-Åsberg depression rating scale (MADRS)

The Montgomery-Åsberg Depression Rating Scale (MADRS) is a standardized rating scale designed to assess symptoms of an ongoing depression [25]. The validity of the scale has been supported in several studies [26]. The MADRS includes 10 phenomena related to depression, and clinicians assess each phenomenon with a rating of 0 to 6.

#### Alcohol use disorders identification test (AUDIT)

The alcohol use disorders identification test (AUDIT) is a 10-item self-report questionnaire developed by a World Health Organization (WHO) collaboration project to assess whether a person is at risk of alcohol-abuse problems and alcohol dependence [27]. Several studies have found that the questionnaire is a reliable and valid measure for identifying alcohol-abuse problems [28].

#### Drug-use disorder identification test (DUDIT)

The drug-use disorder identification test (DUDIT) is an 11-item self-report questionnaire designed to assess whether a person is at risk of drug abuse and drug dependence. Research has shown that this is an effective screening in clinically selected groups for drug-related problems [29].

#### Diagnoses

The diagnoses were reported from the assessment when they first met the crisis resolution team. Some patients had already been diagnosed with a mental illness before they were referred to the crisis resolution team, some patients were diagnosed in their first assessment in the team, and some patients did not report or meet the criteria for a diagnosis within the ICD-10 classification of mental and behavioral disorders [30].

### Procedure

The participants were asked to participate in the study after referral and following their first meeting with clinicians at the hospital. They were all given a written information form and the nature of the study was explained to them. Those who wanted to participate were given a consent form to sign and informed that they would have the right to withdraw at any time.

The clinicians involved in the data collection were all trained to complete the screening interview. This training included a video made by the developers of the CSHF, in addition to an observation of an interview between a researcher and a patient.

### Statistical analysis

**Fig. 1:**
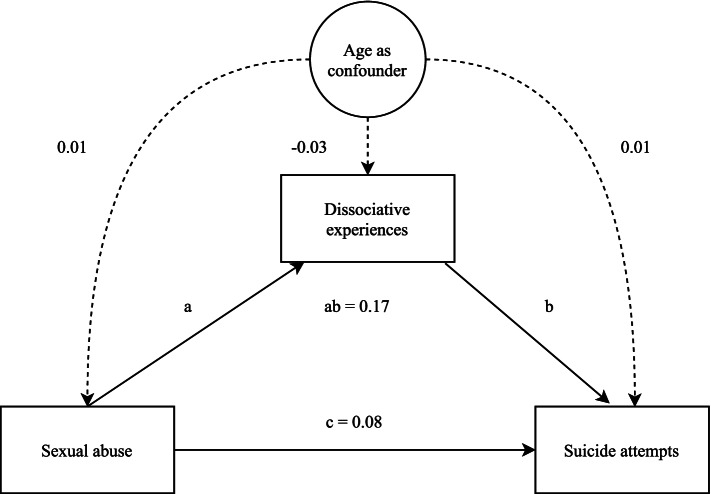
Visual depiction of primary SEM model

Statistical analyses were performed using SPSS [31] and JASP [32]. A descriptive analysis was conducted to compare suicidal behavior in the *sexual abuse* and *no sexual abuse* groups. T-tests and chi-square tests were used where appropriate. Mediation analyses was conducted with Structural Equation Modelling (SEM), following the methods outlined by Woody [33], using 100,000 bootstrapped samples and full-information maximum likelihood to handle missing data. Power analysis following methods described by Liu and Wang (2019) [34] indicated low power (0.50) to detect individual mediation effect (highest expected = 0.30). However, given the potential importance of the study and known difficulties in gathering large samples in the population of suicide attempters [35] we continued the analyses. The primary model (Fig. 1) included sexual abuse as a predictor, dissociation as mediator, and number of suicide attempts as dependent variable. Confounders (age and gender) and other mediators (depression, drug abuse, alcohol abuse) were added in separate models. If confounders or additional mediators increased *R*^2^, these were added to the model. The best fitting model was the original, with the addition of age as a confounder, and only this model is presented. The same model was used to investigate repeated suicide behavior, defined as being in the 80th percentile of number of suicide attempts (> 2 attempts) and being in the 90th percentile of number of suicide attempts (> 4 attempts). Additional information on scale correlations and fits can be found in supplement 1.

## Results

Table 1 shows the characteristics of the patients included in the analyses. The most common diagnostic group represented were affective disorders, neurotic and stress-related disorders, personality disorders, and disorders related to substance use. The most-used psychotropics were hypnotics.

**Table 1:** Characteristics of patients included in the analysis, *N* = 97

	N	%	Mean	SD
Age			35.5	14.0
***Gender***				
Male	44	45		
Female	53	55		
Education in years			13.1	3.4
Depression score			24.8	7.3
***Main diagnosis, ICD-10***				
F10–19 Disorders related to substance use	4	4.1		
F20–29 Disorders related to psychosis	1	1.0		
F30–39 Affective disorders	41	42		
F40–48 Neurotic and stress-related disorders	25	25.8		
F50 Eating disorder	1	1.0		
F60–69 Personality disorders	12	12.3		
F80–89 Developmental disorders	2	2.0		
F90–98 Behavioral and emotional disorders	2	2.0		
*Unspecified diagnosis*	*9*	*9.8*		
***Psychotropics***				
Antidepressant	25	26.6		
Antipsychotics	12	12.8		
Mood stabilizers	12	12.8		
Hypnotics	29	30.9		
Anxiolytics	22	23.4		
Central stimulants	5	5.3		

Table 2 shows the clinical variables for the acute psychiatric patients reporting sexual abuse and no sexual abuse. Patients who had experienced sexual abuse scored significantly higher on the AUDIT and DES. The patients who had experienced sexual abuse were younger when they first had thoughts of suicide and more likely to have self-harmed and attempted suicide in their lifetime. The sexual abuse patient group scored higher on the DES and included more patients with pathological dissociation than patients with no experiences of sexual abuse.

**Table 2:** Clinical variables for acute psychiatric patients reporting sexual abuse and no sexual abuse

N = 97	Sexual abuse ***n*** = 31	No sexual abuse ***n*** = 66	***P*** value	Cohens d
**Gender**			0.08	0.4
Male (n)	10	34		
Female (n)	21	32		
MADRS total score	26.5	24.0	0.14	0.3
AUDIT total score	32.0	11.0	0.01	0.9
DUDIT total score	4.2	3.3	0.59	0.1
DES total score	24.9	14.7	0.02	0.7
DES pathological	66.7%	18.2%	0.01	0.6
**Suicidal behavior**				
Age of first suicide thought	16.8	23.1	0.05	0.4
Self-harm lifetime	1.6	1.3	0.01	0.7
Suicide attempt lifetime	1.8	1.5	0.03	0.5
High lethality suicide attempt	1.4	1.4	0.63	0.1
Total number of suicide attempts	6.7	2.0	0.07	0.4

Table 3 displays the results of the bootstrapped mediation analysis of dissociation as a mediator of sexual abuse and number of suicide attempts, corrected for the confounding effect of age. These analyses revealed that dissociative experiences significantly mediated a substantive proportion of the relationship between sexual abuse and number of suicide attempts (indirect effects = 0.17, 95% CI [0.05, 0.28], proportion mediated = 68%). Dissociative experiences did not significantly mediate the role of sexual abuse as a predictor of making more than 2 suicide attempts (indirect effects = 0.07, 95% CI [0.00, 0.15], proportion mediated = 21%). However, dissociative experiences did significantly mediate the role of sexual abuse as a predictor of making more than 4 suicide attempts (indirect effects = 0.11, 95% CI [0.02, 0.19], proportion mediated = 34%).

**Table 3:** Statistics for mediation analyses

Outcome	Age confound predictor variable (95% CI)	Age confound mediating variable (95% CI)	Age confound outcome variable (95% CI)	Direct effects (95% CI)	Indirect effects (95% CI)	Total effects (95% CI)	R2 Outcome	% mediated
Number of suicide attempts	0.01 (−0.01, 0.02)	−0.03 (−0.04, −0.01)	0.01 (0.01, 0.02)	0.09 (− 0.15, 0.32)	0.17 (0.05, 0.28)	0.25 (0.02, 0.49)	0.24	68%
More than 2 attempts	0.01 (−0.01, 0.02)	− 0.03 (− 0.04, − 0.01)	0.01 (− 0.01, 0.01)	0.26 (0.07, 0.45)	0.07 (0, 0.15)	0.33 (0.15, 0.51)	0.15	21%
More than 4 attempts	0.01 (− 0.01, 0.01)	− 0.03 (− 0.04, − 0.01)	0.01 (0.01, 0.03)	0.21 (0.02, 0.40)	0.11 (0.02, 0.19)	0.32 (0.14, 0.50)	0.19	34%

*Note*. Each row represents a separate mediation model. For all three models Sexual abuse is the predictor and dissociative experiences the mediator, and age is confounding variable. All measures are standardized. “Direct effects” describes the relationship between predictor and outcome that is not explained by mediation. “Indirect effects” describes the relationship between predictor and outcome that is explained by mediation. Total effects is the combination of indirect and direct effects

## Discussion

Dissociation mediated a substantive proportion of the relationship between sexual abuse and number of suicide attempts. As predicted, psychiatric patients who have experienced sexual abuse are more likely to have self-harmed and attempted suicide than patients who have not experienced sexual abuse. Also as predicted, patients in the sexual abuse group reported more pathological dissociation and higher levels of dissociation. The model further identified dissociation as a significant mediating factor of the predictive value of sexual abuse when identifying patients with more than 4 suicide attempts and close to significant mediating effect for patients with more than 2 attempts. A mechanism can be that dissociation heighten vulnerability to stress and this disposition can be a facilitator of helplessness, hopelessness, intolerable stress, and suicidal behavior [36].

The interpersonal theory of suicide [37] explains suicidal behavior as consisting of both the desire to die and the capability to die. The desire to die can arise from thwarted belongingness, perceived burdensomeness, and feelings of hopelessness [38]. The capability to engage in suicidal behavior emerges through habituation and opponent processes after repeated exposure to physical pain or fear-inducing experiences [37]. In the present study, sexual abuse can be understood as an exposure to psychological provocative or fear inducing situations, that could be contributing to a desire to die. Dissociation with its physical disconnection from one’s body could be understood as a facilitator of the habituation to fear that creates courage or fearlessness that builds capability to suicide. Self harm and attempting suicide (and multiple suicide attempts) could be building blocks to suicide capability trough the habituation of pain [39]. An implication of this study is that treatment of dissociation should be included in suicide prevention.

Treatment of dissociative reactions often uses a carefully paced, three-stage process that involves building a therapeutic relationship, increasing safety and stability, developing skills in regulating emotions and managing dissociation, processing traumatic memories, and integrating a sense of self. The final work often includes a focus on developing relationships and life quality. In treatment, one must often revisit the different stages over time [40]. The treatment of patients with dissociative disorders (TOP DD), one of the largest and most geographically diverse studies of treatment of dissociative disorders, followed this phase-stage treatment and found a reduction in dissociation, suicide attempts, non-suicidal self-injury, risky behaviors, and substance use [41].

Self harm and suicide attempt is not always easy to separate into two different categories. If the multi-suicide attempts are more an expression to regulate emotions or a form of communication for help, it could be argued that this form of suicidal behaviour is different from suicide attempt where the intension of suicide is higher. These processes could be difficult to detect and understand correctly in an interview. It is important to address the nuances of suicide intension in assessment of suicide attempt and difficult to measure it correctly [42].

In the analysis, age was found to be a confounding variable for the best fitting model. The confounding effect of age might reflect the frequency of sexual abuse experiences, as well as the number of suicide attempts rising with increasing age. Research has suggested that dissociative experiences decrease with age [43]. It is possible to have less dissociation with age and with more lived life have experienced more abuse and more attempted suicide.

Future studies could include a non-clinical population to achieve a more complete understanding of the relationships between trauma, dissociation, and suicidal behavior. It could be that the occurrence and severity of dissociation in a sample of acute psychiatric patients with suicidal behavior is somewhat different from those in the general population. Research has shown that dissociation is present in a variety of different mental disorders [44]. There is evidence of a robust relationship between disruptions in sleep patterns and dissociation [45] and disrupted sleep is common for most psychiatric patients.

The limitations of this study are the relatively small sample and the *no sexual abuse* group being twice the size of the *sexual abuse* group. In addition, there was no measure of the duration, severity or impact of sexual abuse on life quality or mental health in general for each individual. The severity of sexual abuse could have had an impact on both dissociation and suicidal behavior. The level of psychological strain caused by sexual abuse is relevant to suicide risk and could be included in future studies. It could also be valuable to explore the nuances of suicide capability in the dissociation-suicide attempt link.

## Conclusion

In this study, dissociation mediated a substantive proportion of the relationship between sexual abuse and number of suicide attempts. The results in this study illustrate the importance of assessment and treatment of sexual abuse and trauma-related symptoms such as dissociation in suicide prevention. Dissociation can be a reason why some people act on their suicidal thoughts.

## Supplementary Information

**Additional file 1: S1. Table 1** Scale correlations. **S1. Table 2** Fit indices for model 1. **S1. Table 3** Residual variances model 1. **S1. Table 4** Covariances model 1. **S1. Table 5** Fit indices for model 2. **S1. Table 6** Residual variances model 2. **S1. Table 7** Covariances model 2. **S1. Table 8** Fit indices for model 3. **S1. Table 9** Residual variances model 3. **S1. Table 10** Covariances model 3.

## Data Availability

The dataset analyzed during the current study are available from the corresponding author on reasonable request.

## Abbreviations

CSHF: The Columbia suicide history form
BTQ: The Brief trauma questionnaire
DES: Dissociative experiences scale
MADRS: The Montgomery-Åsberg Depression Rating Scale
AUDIT: The alcohol use disorders identification test
DUDIT: The drug-use disorder identification test
WHO: World Health Organization
SEM: Structural Equation Modelling
TOP DD: The treatment of patients with dissociative disorders
