# Supplementary material for: The effect of sexual abuse and dissociation on suicide attempt
Source: BMC Psychiatry. 2022 Jan 10;22:29. doi: 10.1186/s12888-021-03662-9 (PMC8751353; doi:10.1186/s12888-021-03662-9)
Supplement: Supplementary file 1 — Additional file 1: S1. Table 1 Scale correlations. S1. Table 2 Fit indices for model 1. S1. Table 3 Residual variances model 1. S1. Table 4 Covariances model 1. S1. Table 5 Fit indices for model 2. S1. Table 6 Residual variances model 2. S1. Table 7 Covariances model 2. S1. Table 8 Fit indices for model 3. S1. Table 9 Residual variances model 3. S1. Table 10 Covariances model 3. [file 12888_2021_3662_MOESM1_ESM.docx]

| S1. Table 1 Scale correlations | | | | | | | | | | | | | |
| --- | --- | --- | --- | --- | --- | --- | --- | --- | --- | --- | --- | --- | --- |
| Variable | |  | | sexual abuse | | dissociative experiences | | suicide attempts | | suicide attempts > 2 | | suicide attempts > 4 | |
| sexual abuse |  | Pearson's r |  | — |  |  |  |  |  |  |  |  |  |
| dissociative experiences |  | Pearson's r |  | 0.31 | ** | — |  |  |  |  |  |  |  |
| suicide attempts |  | Pearson's r |  | 0.19 |  | 0.34 | *** | — |  |  |  |  |  |
| suicide attempts > 2 |  | Pearson's r |  | 0.32 | ** | 0.31 | ** | 0.39 | *** | — |  |  |  |
| suicide attempts > 4 |  | Pearson's r |  | 0.31 | ** | 0.31 | ** | 0.58 | *** | 0.61 | *** | — |  |
|  | | | | | | | | | | | | | |
| * p < .05, ** p < .01, *** p < .001 | | | | | | | | | | | | | |

#

# Supplement to mediation model predicting number of attempts

| S1. Table 2 Fit indices for model 1 | | | |
| --- | --- | --- | --- |
| Metric | | Value | |
| Comparative Fit Index (CFI) |  | 1.00 |  |
| Tucker-Lewis Index (TLI) |  | 1.00 |  |
| Akaike (AIC) |  | 1071.00 |  |
| Bayesian (BIC) |  | 1110.09 |  |
| Root mean square error of approximation (RMSEA) |  | 0.00 |  |
| RMSEA 90% CI lower bound |  | 0.00 |  |
| RMSEA 90% CI upper bound |  | 0.00 |  |

##

##

| S1. Table 3 Residual variances model 1 | | | | | | | | | | | | | | | |
| --- | --- | --- | --- | --- | --- | --- | --- | --- | --- | --- | --- | --- | --- | --- | --- |
|  | | | | | | | | | | | | 95% Confidence Interval | | | |
| Variable | |  | | Estimate | | Std. Error | | z-value | | p | | Lower | | Upper | |
| suicide attempts |  |  |  | 0.75 |  | 0.09 |  | 8.18 |  | 2.22e -16 |  | 0.57 |  | 0.93 |  |
| dissociative experiences |  |  |  | 0.84 |  | 0.12 |  | 7.02 |  | 2.24e -12 |  | 0.60 |  | 1.07 |  |
| sexual abuse |  |  |  | 1.02 |  | 0.15 |  | 7.01 |  | 2.43e -12 |  | 0.73 |  | 1.31 |  |
| alder |  |  |  | 201.24 |  | 0.00 |  |  |  |  |  | 201.24 |  | 201.24 |  |

##

##

###

| S1. Table 4 Covariances model 1 | | | | | | | |
| --- | --- | --- | --- | --- | --- | --- | --- |
| suicide attempts | | dissociative experiences | | sexual abuse | | alder | |
| 1.00 |  |  |  |  |  |  |  |
| 0.47 |  | 1.10 |  |  |  |  |  |
| 0.26 |  | 0.37 |  | 1.02 |  |  |  |
| 0.45 |  | -5.08 |  | 0.22 |  | 201.24 |  |

##

#

#

| S1. Table 5 Fit indices for model 2 | | | |
| --- | --- | --- | --- |
| Metric | | Value | |
| Comparative Fit Index (CFI) |  | 1.00 |  |
| Tucker-Lewis Index (TLI) |  | 1.00 |  |
| Akaike (AIC) |  | 1070.22 |  |
| Bayesian (BIC) |  | 1109.31 |  |
| Root mean square error of approximation (RMSEA) |  | 0.00 |  |
| RMSEA 90% CI lower bound |  | 0.00 |  |
| RMSEA 90% CI upper bound |  | 0.00 |  |

##

##

| S1. Table 6 Residual variances model 2 | | | | | | | | | | | | | | |
| --- | --- | --- | --- | --- | --- | --- | --- | --- | --- | --- | --- | --- | --- | --- |
|  | | | | | | | | | | | | 95% Confidence Interval | | |
| Variable | |  | | Estimate | | Std. Error | | z-value | | p | | Lower | | Upper |
| suicide attempts > 2 |  |  |  | 0.84 |  | 0.10 |  | 8.64 |  | 0.00 |  | 0.65 |  | 1.03 |
| dissociative experiences |  |  |  | 0.77 |  | 0.11 |  | 6.97 |  | 3.24e -12 |  | 0.55 |  | 0.99 |
| sexual abuse |  |  |  | 0.99 |  | 0.14 |  | 7.02 |  | 2.20e -12 |  | 0.72 |  | 1.27 |
| alder |  |  |  | 201.24 |  | 0.00 |  |  |  |  |  | 201.24 |  | 201.24 |

##

##

| S1. Table 7 Covariances model 2 | | | | | | | |
| --- | --- | --- | --- | --- | --- | --- | --- |
| suicide attempts > 2 | | dissociative experiences | | sexual abuse | | alder | |
| 0.99 |  |  |  |  |  |  |  |
| 0.30 |  | 0.99 |  |  |  |  |  |
| 0.33 |  | 0.31 |  | 0.99 |  |  |  |
| -0.98 |  | -4.92 |  | 0.38 |  | 201.24 |  |

##

#

#

| S1. Table 8 Fit indices for model 3 | | | |
| --- | --- | --- | --- |
| Metric | | Value | |
| Comparative Fit Index (CFI) |  | 1.00 |  |
| Tucker-Lewis Index (TLI) |  | 1.00 |  |
| Akaike (AIC) |  | 1066.20 |  |
| Bayesian (BIC) |  | 1105.29 |  |
| Root mean square error of approximation (RMSEA) |  | 0.00 |  |
| RMSEA 90% CI lower bound |  | 0.00 |  |
| RMSEA 90% CI upper bound |  | 0.00 |  |

##

| S1. Table 9 Residual variances model 3 | | | | | | | | | | | | | | |
| --- | --- | --- | --- | --- | --- | --- | --- | --- | --- | --- | --- | --- | --- | --- |
|  | | | | | | | | | | | | 95% Confidence Interval | | |
| Variable | |  | | Estimate | | Std. Error | | z-value | | p | | Lower | | Upper |
| suicide attempts > 4 |  |  |  | 0.81 |  | 0.10 |  | 8.45 |  | 0.00 |  | 0.62 |  | 0.99 |
| dissociative experiences |  |  |  | 0.77 |  | 0.11 |  | 6.98 |  | 3.03e -12 |  | 0.55 |  | 0.99 |
| sexual abuse |  |  |  | 0.99 |  | 0.14 |  | 7.02 |  | 2.26e -12 |  | 0.72 |  | 1.27 |
| alder |  |  |  | 201.24 |  | 0.00 |  |  |  |  |  | 201.24 |  | 201.24 |

##

###

| S1. Table 10 Covariances model 3 | | | | | | | |
| --- | --- | --- | --- | --- | --- | --- | --- |
| suicide attempts > 4 | | dissociative experiences | | sexual abuse | | alder | |
| 1.00 |  |  |  |  |  |  |  |
| 0.33 |  | 1.01 |  |  |  |  |  |
| 0.32 |  | 0.32 |  | 0.99 |  |  |  |
| 1.02 |  | -5.23 |  | 0.11 |  | 201.24 |  |
